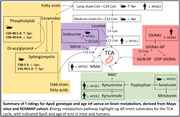# ApoE in brain mitochondria and energetics: From mice to human

**DOI:** 10.1002/alz.089796

**Published:** 2025-01-03

**Authors:** Kamil Borkowski, Nuanyi Liang, Matthias Arnold, Guojun Bu, Rima Kaddurah‐Daouk, Na Zhao, Takahisa Kanekiyo

**Affiliations:** ^1^ University of California, Davis, Davis, CA USA; ^2^ Department of Psychiatry and Behavioral Sciences, Duke University, Durham, NC USA; ^3^ Mayo Clinic, Jacksonville, FL USA; ^4^ Duke University Medical Center, Durham, NC USA

## Abstract

**Background:**

Alzheimer’s disease (AD) risk and progression are significantly influenced by ApoE genotypes, with ApoE4 increasing and ApoE2 decreasing the susceptibility compared to ApoE3. Understanding metabolic pathways affected by ApoE genotypes will help decipher disease development and identify new therapeutic targets.

**Method:**

This study investigates the impact of ApoE genotypes on aging brain metabolic trajectories using human ApoE‐targeted replacement mice. Applying Biocrates P180 targeted metabolomics platform, we analyzed the metabolic impact of ApoE2/2, ApoE3/3, and ApoE4/4 on fatty acid β‐oxidation, amino acids, and phospholipids, which are known to be altered in AD. Furthermore, we compared our rodent model results with human dorsolateral prefrontal cortex data from the Religious Orders Study/Memory and Aging Project (ROS‐MAP).

**Result:**

We found aging mice carrying ApoE2/2 had altered branch‐chain amino acid metabolism and increased C5 acylcarnitine and its ratio to precursor isoleucine, pointing towards increased β‐oxidation and branched‐chain amino acid (BCAA) utilization. Furthermore, ROS‐MAP data revealed the ApoE2 genotype affects similar areas of metabolism in humans. Additionally, our data provide comprehensive insight into age‐related metabolic changes of the current mice model, independent of ApoE genotype, among phospholipids, sphingomyelins, amino acids, and biogenic amines. Many of the observed differences are also known aging markers in humans, with a connection to cognition and Alzheimer’s disease.

**Conclusion:**

Together, these results suggest a potential involvement of ApoE2/2 genotype in energy metabolism and characterize the current mice model for further study of ApoE in AD, brain aging, and brain BCAA utilization for energy.